# Cholesterol-lowing effect of taurine in HepG2 cell

**DOI:** 10.1186/s12944-017-0444-3

**Published:** 2017-03-16

**Authors:** Junxia Guo, Ya Gao, Xuelian Cao, Jing Zhang, Wen Chen

**Affiliations:** 1 Food Science Department, College of Biochemical Engineering, Beijing Union University, Fatou west 18#, Chaoyang District, Beijing, 100023 People’s Republic of China; 2 Beijing Key Laboratory of Bioactive Substances and Functional Foods, Beijing Union University, Beijing, 100191 People’s Republic of China

**Keywords:** Taurine, Cholesterol, Bile acids, HepG2 cell

## Abstract

**Background:**

A number of studies indicate that taurine promotes cholesterol conversion to bile acids by upregulating CYP7A1 gene expression. Few in vitro studies are concerned the concentration change of cholesterol and its product of bile acids, and the molecular mechanism of CYP7A1 induction by taurine.

**Methods:**

The levels of intracellular total cholesterol (TC), free cholesterol (FC), cholesterol ester (EC), total bile acids (TBA) and medium TBA were determined after HepG2 cells were cultured for 24/48 h in DMEM supplemented with taurine at the final concentrations of 1/10/20 mM respectively. The protein expressions of CYP7A1, MEK1/2, c-Jun, p-c-Jun and HNF-4*α* were detected.

**Results:**

Taurine significantly reduced cellular TC and FC in dose —and time-dependent ways, and obviously increased intracellular/medium TBA and CYP7A1 expressions. There was no change in c-Jun expression, but the protein expressions of MEK1/2 and p-c-Jun were increased at 24 h and inhibited at 48 h by 20 mM taurine while HNF4α was induced after both of the 24 h and 48 h treatment.

**Conclusion:**

Taurine could enhance CYP7A1 expression by inducing HNF4α and inhibiting MEK1/2 and p-c-Jun expressions to promote intracellular cholesterol metabolism.

## Background

Taurine (2-aminoethanesulfonic acid) is a sulfur-containing amino acid mainly obtained through diet in humans as only small amounts can be synthesized endogenously [1]. A great number of studies have revealed that taurine has cholesterol-lowering effect since Tsuji et al. published their experimental data in 1979 [2–4]. Furthermore, many studies indicate that the cholesterol-lowering effect of taurine is due to the increased biotransformation of cholesterol to bile acids in the liver and the subsequent excretion of bile acids in feces [5–7].

Cholesterol conversion or bile acids biosynthesis is critically regulated in order to maintain cholesterol or bile acids homeostasis in the body. Cholesterol 7α-hydroxylase (CYP7A1), the rate-limiting enzyme in the major bile acids synthetic pathway, is widely reported to be regulated by several nuclear receptors at the level of gene transcription [8–10]. It has been confirmed that liver X receptor-α (LXRα) is considered as a positive regulator of CYP7A1 transcription, and hepatocyte nuclear factor 4α (HNF4α) and liver receptor homolog-1 (LRH-1) are essential factors for basal level expression of CYP7A1 [11, 12]. It has also been pointed out that there are two pathways to feedback repress CYP7A1 transcription, one being the bile acids receptor---farnesoid X receptor (FXR)-dependent which interacts with HNF4α and LRH-1 via atypical nuclear receptor small heterodimer partner-1 (SHP-1) induced by FXR, and the other being the FXR-independent pathway which involves many factors such as the tumor necrosis factor (TNF) receptor, the mitogen-activated protein kinase/c-Jun N-terminal kinase (MAPK/JNK), the mitogen-activated protein kinase/extracellular signal regulated kinase (MAPK/ERK, MEK) signal transduction etc. [13–15].

Many studies on the hypercholesterolemia rat and mouse have revealed that taurine increases the mRNA expression and activity of CYP7A1 [5, 6, 16, 17], and the same improvement has also been observed via the in vitro experiment [7]. The in vitro studies have focused on the upregulating-effect of taurine on CYP7A1 mRNA level, but the regulatory mechanism and the changes of cellular bile acids, the products of cholesterol degradation, have not been discussed. Moreover, the molecular mechanism of CYP7A1 induction by taurine has rarely been discussed although the regulatory pathway of CYP7A1 and its relative nuclear receptors or factors have been studied extensively. In 2006, Lam et al. reported that taurine almost did not alter the mRNA levels of LXRα, LRH-1, FXR and SHP-1 of mice with high cholesterol or cholesterol/sodium cholate diets, although CYP7A1 mRNA level was significantly decreased by cholesterol/sodium cholate diet and then two-fold increased by taurine supplementation [18]. Their results suggested that FXR was indeed activated by cholesterol/sodium cholate diet and then functioned as a down-regulator to CYP7A1 in spite of no change of mRNA level, and that taurine might stimulate CYP7A1 expression not by repressing FXR-dependent pathway but by FXR-independent pathway.

In the present study, HepG2 cell line derived from human hepatoma cell was used to investigate the dose- and time-dependent effects of taurine with a focus on the change of CYP7A1 expression and the concentration of cholesterol and bile acids. In addition, the expressions of several important factors involved in FXR-independent pathway such as MEK1/2, phosphorylated c-Jun (p-c-Jun) and HNF4α etc. were determined to discuss the preliminary molecular mechanism of CYP7A1 induction by taurine.

## Methods

### Reagents

The cell culture reagents were obtained from Hyclone (Logan, USA) and the fetal bovine serum (FBS) was obtained from Gibco (New York, USA). The taurine, cholesterol, cholesterol esterase, cholesterol oxidase, horseradish peroxidase and sodium taurocholate hydrate were purchased from Sigma (St. Louis, MO, USA). The PowerOpti-ECL Western blotting detection reagent was purchased from GenView (Florida, USA). The primary (anti-CYP7A1, sc-25536; anti-c-jun, sc-1694; anti-p-c-jun, sc-7980-R; and anti–HNF4α, sc-8987) and secondary (anti-rabbit immunoglobulin G, sc-2004) antibodies were acquired from Santa Cruz Biotechnology (Santa Cruz, CA, USA). The primary anti-MEK1/2 antibody (D1A5) was obtained from Cell Signaling Technology (Beverly, MA, USA). The primary anti-β-actin antibody was obtained from GenView (Florida, USA). The BCA Protein Assay Kit, Cell Lysis Buffer and PMSF were purchased from Beyotime (Nanjing, Jiangsu, China). All the other chemicals were purchased from Dingguo Changsheng Biotechnology Co. Ltd (Beijing, China).

### Cell culture and treatment

The HepG2 cell line obtained from Cell Resource Center (School of Basic Medicine Peking Union Medical College) were routinely cultured in DMEM supplemented with 10% heat-inactivated fetal calf serum, 2 mM L-glutamine, 100U/ml penicillin and 100ug/ml streptomycin. The cells were grown in the 75 mm^2^culture flasks at 37 °C in a humidified atmosphere of 95% air and 5% CO_2_ before treatment. For the experiment, the cells were plated out in 6-well plates and incubated in media containing taurine (0, 1, 10 and 20 mM) for 24 h or 48 h. The cells with 0 mM taurine treatment were taken as control and were given PBS instead of taurine. Each treatment was carried out at least in triplicate.

The results of our preliminary experiments showed that about 90% of the cells survived in the medium with final concentration of 20 mM taurine, while less than 70% survived with 30 mM. Thus, 20 mM taurine was determined as the largest concentration in present experiments.

### Cellular cholesterol quantification

The intracellular cholesterol was determined by the description of Omodeo with few modifications [19]. Briefly, after being washed for 3 times with cold PBS, the cells were treated with 1 mL hexane/isopropanol (2:1, v:v) for 30 min at room temperature. The organic solvent was transferred to the test tubes, then the wells were washed with an additional 1 mL hexane/isopropanol, and the washing solutions were also transferred to the corresponding test tubes. The organic solvent was removed under nitrogen, and the lipids were resuspended in 130 μL isopropanol with 10% TritonX-100. The cellular total cholesterol (TC) and free cholesterol (FC) content were measured as follows: 50 μL of the lipid samples was treated with TC or FC working solution shown in Table 1 for 30 min at 37 °C and optical density was determinate at 500 nm using a μQuant microplate spectrophotometers (Bio-Tek instruments INC, USA). The cellular ester cholesterol (EC) was calculated by TC minus FC.

**Table 1:** Composition of working solutions for cholesterol detection

Chemicals	TC (μL)	FC (μL)
Dipotassium hydrogen phosphate (0.1 M)	192	198
Cholesterol oxidase (5U/mL)	6	6
Horse radish peroxidase (50U/mL)	6	6
Sodium taurocholate hydrate (20 mM)	15	15
TritonX-100(1%)	15	15
4-aminoantipyrine (5.5 mM)	45	45
Phenol (280 mM)	15	15
Cholesterol esterase (25U/mL)	6	0
Total volume (μL)	300	300

After the cell lipids were extracted by the organic solvent, the remainders were lysed in Cell Lysis Buffer and then the protein concentration was determined by the BCA Protein Assay Kit.

### Intracellular and medium bile acids quantification

The HepG2 cells and their medium were separately collected. The cells were lysed with PBS containing 2% Triton X-100 for 45 min and supernatant were obtained by centrifugation at 10,000 rpm for 10 min. Then the total bile acids (TBA) in the supernatant and in the medium were quantified respectively by using enzyme colorimetric method by TBA kit (Nanjing Jiancheng technologies Co., China) following the manufacturer’s instructions. The cellular protein concentration was also determined by BCA Protein Assay Kit.

### Immunoblotting assay

The HepG2 cells were lysed in ice-cold lysis buffer containing 10 mM Tris–HCl (pH 7.4), 0.1 M EDTA, 10 mM NaCl, 0.5% Triton X-100 and PMSF. The homogenates were clarified by centrifugation at 12,000 rpm for 10 min at 4 °C, and the protein concentration was determined by the BCA Protein Assay Kit. The samples with the same amount of protein were boiled in the sample buffer (5% β-mercaptoethanol) for 5 min, and then were separated by SDS-PAGE and blotted onto a PVDF membrane (Millipore, USA). After the nonspecific protein binding was blocked by incubation in PBS containing 5% nonfat dry milk, the membranes were incubated with the primary antibodies overnight at 4 °C which were diluted in the blocking buffer as follows: anti-CYP7A1, 1:1000; anti-c-jun, 1:800; anti-p-c-jun, 1:1000; anti-MEK1/2, 1:2000; anti-HNF4α, 1:800. Subsequently the membranes were incubated with 1:2000 dilutions of secondary antibody for 1 h, and immunoreactive bands were detected by using an ECL western blot detection reagent and exposed to the chemiluminescence film of high performance for 1 ~ 5 min. Finally, the immunoblots were scanned and quantified by using a Gel imaging system (GE healthcare, USA).

### Statistical analysis

All the data were expressed as the mean ± standard error, and the statistical analysis was performed by one-way ANOVA. The differences were considered to be statistically significant at *p* < 0.05.

## Results

### Taurine reduced cellular cholesterol and increased bile acids

The in vivo experiments have confirmed that taurine significantly decreases the serum and liver cholesterol levels and increases the fecal bile acids excretion [4, 5, 16, 17]. In the present in vitro experiment HepG2 cells were incubated with taurine for 24 h or 48 h and the changes of TC, FC, EC and TBA were determined.

As shown in Table 2, taurine significantly reduced the cellular TC, FC and EC after the 24 h or 48 h culture, and the decreases of TC and FC were apparently dose- and time-dependent with taurine. Table 3 shows that the intracellular TBA levels increased by taurine in a dose-dependent way after 24 h incubation, while the TBA concentration in medium also distinctly enhanced but reached the peak at 10 mM taurine treatment. This implied that time was needed for the excretion of the intracellular TBA synthesized from cholesterol to the culture medium. After the culture for 48 h, the intracellular TBA increased marginally and reached a peak at 10 mM taurine whereas the medium TBA improved in a dose-dependent way. Additionally, both of the intracellular and the medium TBA levels were further increased by 1 mM taurine comparison with that of 24 h-incubation. This suggested that 48 h of culture seemed to be the time required for the intracellular TBA production and its excretion into the medium. All the above results indicated that 20 mM taurine most effectively reduced the intracellular cholesterol and increased the intracellular and medium bile acids after 48 h treatment.

**Table 2:** The effect of taurine on cellular cholesterol level (μg/mg · pro)

	Control	Taurine		
		1 mM	10 mM	20 mM
		Culture for 24 h (*n =* 5)		
TC	150.9 ± 7.7	140.8 ± 6.3	134.8 ± 5.3*	127.3 ± 5.9*
FC	105.2 ± 7.0	99.2 ± 6.0	93.5 ± 5.3*	86.9 ± 6.2*
EC	45.6 ± 3.1	41.6 ± 3.8	41.3 ± 2.3	40.4 ± 2.3*
		Culture for 48 h (*n =* 6)		
TC	153.8 ± 2.7	132.7 ± 3.6*	119.6 ± 3.7*	112.1 ± 4.6*
FC	111.0 ± 4.8	98.3 ± 5.8*	85.3 ± 3.6*	72.5 ± 4.5*
EC	42.8 ± 4.4	34.4 ± 5.1*	34.3 ± 6.4*	39.5 ± 5.1

*Compared with control, *P <* 0.05

**Table 3:** The effect of taurine on intracellular and medium TBA levels

	Control	Taurine		
		1 mM	10 mM	20 mM
		culture for 24 h (*n =* 5)		
Intracellular (μmol/g · pro)	1.05 ± 0.06	1.83 ± 0.07*	2.32 ± 0.05*	2.88 ± 0.11*
Medium (μmol/L)	0.67 ± 0.07	1.37 ± 0.07*	2.72 ± 0.03*	2.27 ± 0.09*
		culture for 48 h (*n =* 6)		
Intracellular (μmol/g · pro)	1.55 ± 0.03	3.24 ± 0.07*	3.46 ± 0.07*	3.03 ± 0.04*
Medium (μmol/L)	1.28 ± 0.03	1.95 ± 0.07*	2.34 ± 0.03*	2.47 ± 0.02*

*Compared with control, *P <* 0.05

### Taurine induced CYP7A1 protein expression

**Fig. 1:**
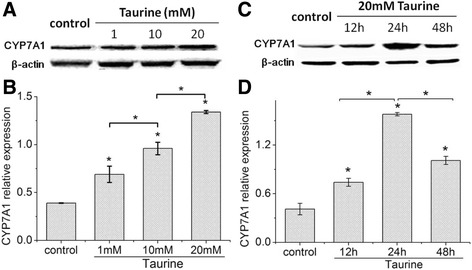
Effect of taurine on CYP7A1 protein expression in HepG2 cells. **a** and **b**, HepG2 cells were treated with taurine (1, 10, 20 mM) for 24 h (*n =* 6), showed that CYP7A1 protein levels significantly increased in a dose-dependent way after 24 h taurine treatment. **c** and **d**, HepG2 cells were treated in 20 mM taurine for 12 h, 24 h and 48 h (*n =* 3), showed CYP7A1 expression improved and reached a peak at 24 h, and the level of 48 h still significantly increased compared with that of control and 12 h. *P < 0.05 compared with control or corresponding group

The dose- and time-relationship between taurine and the CYP7A1 protein expression in HepG2 cells were analyzed by immunoblotting, and β-actin was used as the endogenous control. Figure 1 A/B showed that CYP7A1 protein levels significantly increased in a dose-dependent way after 24 h taurine treatment. Figure 1 C/D showed CYP7A1 protein levels of HepG2 cells treated with 20 mM taurine for 12 h, 24 h and 48 h. The results demonstrated that CYP7A1 expression improved and reached a peak on 24 h, and the level of 48 h still significantly increased compared with that of the control and 12 h.

### Taurine affected several key factors associated with CYP7A1 expression

**Fig. 2:**
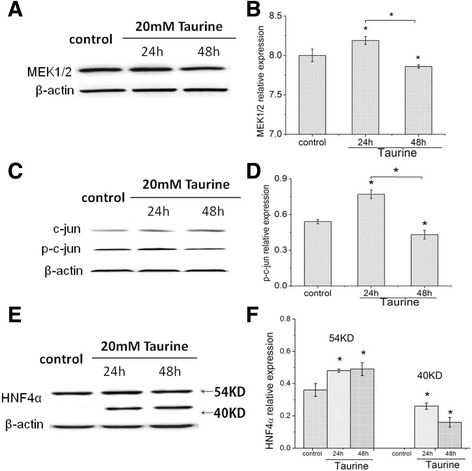
Effect of taurine on several key factors associated with CYP7A1 expression in HepG2 cells. HepG2 cells were incubated with 20 mM taurine for 24 h and 48 h. Fig **a**, **b** and **c**, **d** respectively showed the protein expressions of MEK1/2 and p-c-jun in HepG2 cells were promoted at 24 h and inhibited at 48 h by 20 mM taurine treatment. Fig **e**, **f** showed that HNF4α was dramatically induced at both of 24 h and 48 h. The values represent the mean ± SE (*n =* 3). *P < 0.05 compared with the control and other corresponding group

Several key moleculars involved in the FXR-independent pathway of CYP7A1 regulation were measured. Figure 2 A/B and C/D showed that the protein expression of MEK1/2 and p-c-jun in HepG2 cells were promoted at 24 h and inhibited at 48 h by 20 mM taurine treatment. This suggested that the synthesized bile acids activated MEK1/2 and phosphorylated c-Jun in the early period of cholesterol degradation, and subsequently taurine suppressed MEK1/2 and p-c-Jun expression with prolonged incubating time. Figure 2 E/F indicated that the full-length expression of HNF4α with 54 kDa molecular weight was dramatically induced at both 24 h and 48 h since it has been reported to be essential for CYP7A1 expression, and the truncated HNF-4α N-terminal with 40 kDa molecular weight was also significantly increased accompanying the increase of full length HNF4α by taurine treatment although it was less in control. All the above results indicated that taurine may increase CYP7A1 expression by promoting HNF4α and repressing MEK1/2 and p-c-Jun.

## Discussions

Cholesterol conversion to bile acids in the liver plays a vital role for the elimination of cholesterol, which is one of the main factors regulating cholesterol homeostasis in the body. The first and rate-limiting reaction in the major bile acid synthetic pathway is the 7α-hydroxylation of cholesterol, which is catalyzed by CYP7A1 [20, 21].

Many in vivo studies with hypercholesterolemia rat, mouse and hamster reveal that the cholesterol-lowering effect of taurine is carried out by enhancing CYP7A1 activity or gene expression and fecal bile acid excretion [5, 6, 16, 17], while in vitro researches are few. Yanagita T et al. noted that the cellular cholesterol levels decreased and synthesis of cholesterol ester was inhibited when the HepG2 cells were incubated in DMEM medium with taurine for 24 h [22]. Lam NV et al. reported that taurine significantly increased CYP7A1 mRNA level and promoted cholesterol degradation regardless of whether the HepG2 cells were cultured under the conditions of high cholesterol or not [7], and Hoang HM et al. indicated that CYP7A1 mRNA levels increased 3 times when the H4IIE cells were treated with taurine [23]. The above in vitro studies focused on the effect of taurine on CYP7A1 mRNA level, and the changes of cellular bile acids, the products of cholesterol degradation, were not discussed.

In the present study, the CYP7A1 expression, cellular cholesterol and bile acids were determined in order to comprehensively discuss the effect of taurine on cholesterol metabolism. The results of dose- and time-relationship between taurine and CYP7A1 protein expression showed that CYP7A1 expression significantly increased in a positive dose-dependent way after 24 h treatment, and reached a peak at 24 h when the cell was cultured with 20 mM taurine for 12/24/48 h. The results of cholesterol and bile acids showed that taurine significantly reduced cellular TC and FC in dose- and time-dependent manner, increased intracellular TBA level in dose-dependent way after 24 h treatment and enhanced the medium TBA in dose-dependent way after 48 h culture. Moreover, both of the intracellular and medium TBA levels of 48 h culture were further improved by 1 mM taurine compared with that of the 24 h culture. This suggested that 48 h of culture seemed to be the time needed for the intracellular TBA production and its excretion into medium. All the above results indicate that taurine can enhance CYP7A1 expression and subsequently promote intracellular cholesterol catabolism, and the decrease of cholesterol is associated with taurine concentration and action duration, showing a superior cholesterol lowering effect at 48 h to that at 24 h.

It has been clear that several nuclear receptors play key roles in CYP7A1 regulation to maintain cholesterol/bile acid homeostasis. HNF-4*α*, an orphan member of the nuclear receptor superfamily, is an important regulator of CYP7A1 gene expression as demonstrated by in vitro promoter analysis and by characterization of mice with a liver-specific deficiency of HNF-4*α* or LRH-1 deficiency [24, 25]. The clinical study showed the expression of HNF4α was significantly increased in human liver biopsies from patients treated with cholestyramine in parallel with markedly increased expression of CYP7A1, indicating that HNF4α may also regulate the expression of CYP7A1 in humans [26]. Therefore, HNF4α expression was investigated in the present study, and the data showed that it was induced by taurine after both of the 24 h and 48 h treatment, suggesting that the increase of HNF4α protein level results in an induction of CYP7A1 expression and bile acids synthesis.

It has also been pointed out that there are two pathways to feedback repress CYP7A1 transcription, one being FXR-dependent which interacts with HNF4α and LRH-1 via atypical nuclear receptor SHP-1 induced by FXR, and the other being FXR-independent pathway which involves many factors such as TNF receptor, MAPK/JNK signal transduction, etc. [13–15]. Studies not only suggested that TNFα inhibit HNF4α trans-activating activity and CYP7A1 gene transcription via the MAPK/ERK (MEK) signaling pathway, but also showed that bile acids can activate the c-Jun N-terminal kinase (JNK)/c-Jun pathway to phosphorylate c-Jun which combines with HNF4α to inhibit the CYP7A1 gene [14, 27, 28]. Furthermore, Lam et al. reported that taurine did not alter the mRNA levels of FXR and SHP-1 of mice with high cholesterol diet although the CYP7A1 mRNA level was significantly increased [18], and Das J. indicated that taurine can decrease JNK activity by repressing the phosphorylation of PKCδ in NaAsO_2_ exposure rat’s liver [29]. Therefore, MEK1/2, c-Jun and p-c-Jun expression were investigated in the present study. The data showed there was no apparent change in c-Jun expression, but the protein expression of MEK1/2 and p-c-jun were increased at 24 h and inhibited at 48 h by 20 mM taurine. This suggested that the synthesized bile acids activated MEK1/2 and phosphorylated c-Jun in the early period of cholesterol degradation, and subsequently taurine suppressed MEK1/2 and p-c-Jun expression with prolonged treatment time, and finally relieved the inhibition of bile acid to CYP7A1 gene expression.

## Conclusion

In conclusion, the present study shows that taurine promotes the bioconversion of cholesterol to bile acids in the HepG2 cell, and the decrease of cholesterol or the increase of bile acids is associated with taurine concentration and action duration, showing a superior effect at 48 h to that at 24 h. The results in our study indicate that taurine could enhance CYP7A1 expression by inducing HNF4α and inhibiting MEK1/2 and p-c-Jun to accelerate intracellular cholesterol metabolism. Further experiments need to be done to understand the detailed signaling pathways of CYP7A1 up-regulation by taurine.

## Abbreviations

CYP7A1: Cholesterol 7α-hydroxylase
EC: Ester cholesterol
ERK: Extracellular signal regulated kinase
FC: Free cholesterol
FXR: Farnesoid X receptor
HNF4α: Hepatocyte nuclear factor 4α
LRH-1: Liver receptor homolog-1
LXRα: Liver X receptor-α
MAPK/JNK: Mitogen-activated protein kinase/c-Jun N-terminal kinase
MEK: MAPK/ERK kinase
p-c-Jun: Phosphorylated c-Jun
SHP-1: Small heterodimer partner-1
TBA: Total bile acids
TC: Total cholesterol

## References

[1] Lambert IH, Kristensen DM, Holm JB, Mortensen OH (2015). Physiological role of taurine---from organism to organelle. Acta Physiol (Oxf).

[2] Tsuji K, Seki T, Iwao H (1979). Cholesterol-lowering effects of taurine and sulfur- containing amino acids in serum and liver of rats. Sulfur-Containing Amino Acids.

[3] Mochizuki H, Takido J, Yokogoshi H (1999). Improved suppression by dietary taurine of the fecal excretion of bile acids from hypothyroid rats. Biosci Biotechnol Biochem.

[4] Chen W, Matuda K, Nishimura N, Yokogoshi H (2004). The effect of taurine on cholesterol degradation in mice fed a high-cholesterol diet. Life Sci.

[5] Yokogoshi H, Oda H (2002). Dietary taurine enhances cholesterol degradation and reduces serum and liver cholesterol concentrations in rats fed a high-cholesterol diet. Amino Acids.

[6] Murakami S, Fujita M, Nakamura M, Sakono M, Nishizono S, Sato M, Imaizumi K, Mori M, Fukuda N (2016). Taurine ameliorates cholesterol metabolism by stimulating bile acid production in high cholesterol-fed rats. Clin Exp Pharmacol Physiol.

[7] Lam NV, Chen W, Suruga K, Nishimura N (2006). Enhancing effect of taurine on CYP7A1 mRNA expression in HepG2 cells. Amino Acids.

[8] Janowski BA, Willy PJ, Devi TR, Falck JR, Mangelsdorf DJ (1996). An oxysterol signalling pathway mediated by nuclear receptor LXR alpha. Nature.

[9] Chiang JY, Kimmel R, Stroup D (2001). Regulation of cholesterol 7α-hydroxylase gene (CYP7A1) transcription by the liver orphan receptor (LXRα). Gene.

[10] Li T, Chiang JY (2014). Bile acid signaling in metabolic disease and drug therapy. Pharmacol Rev.

[11] Peet DJ, Truly SD, Ma W, Janowski BA, Lobaccaro J-MA, Hammer RE, Mangelsdorf DJ (1998). Cholesterol and bile acid metabolism are impaired in mice lacking the nuclear oxysterol receptor LXR-α. Cell.

[12] Tavares-Sanchez OL, Rodriguez C, Gortares-Moroyoqui P, Estrada MI (2015). Hepatocyte nuclear factor-4α, a multifunctional nuclear receptor associated with cardiovascular disease and cholesterol catabolism. Int J Environ Health Res.

[13] Goodwin B, Jones SA, Price RR, Watson MA, McKee DD (2006). A regulatory cascade of the nuclear receptors FXR, SHP-1 and LRH-1 represses bile acid biosynthesis. Mol Cell.

[14] Li T, Jahan A, Chiang JY (2006). Bile acids and cytokines inhibit the human cholesterol 7α- hydroxylase gene via the JNK/cjun pathway in human liver cells. Hepatology.

[15] Chiang JYL (2009). Bile acid: regulation of synthesis. J Lipid Res.

[16] Chang YY, Chou CH, Chiu CH, Yang KT (2011). Preventive effects of taurine on development of hepatic steatosis induced by a high-fat/cholesterol dietary habit. J Agric Food Chem.

[17] Chen W, Suruga K, Nishimura N, Gouda T (2005). Comparative regulation of major enzymes in bile acids biosynthesis pathways by cholesterol, cholic acid and taurine in mice and rats. Life Sci.

[18] Lam NV, Chen W, Suruga K, Nishimura N (2006). Effects of taurine on mRNA levels of nuclear receptors and factors involved in cholesterol and bile acid homeostasis in mice. Adv Exp Med Biol.

[19] Omodeo Salè F, Marchesini S, Fishman PH, Berra B (1984). A sensitive enzymatic assay for determination of cholesterol in lipid extracts. Anal Biochem.

[20] Cohen JC (1999). Contribution of cholesterol 7alpha-hydroxylase to the regulation of lipoprotein metabolism. Curr Opin Lipidol.

[21] Chiang JY (1998). Regulation of bile acid synthesis. Front Biosci.

[22] Yanagita T, Han SY, Nagao K, Kitajima H, Murakami S (2008). Taurine reduces the secretion of apolipoprotein B100 and lipids in HepG2 cells. Lipids Health Dis.

[23] Hoang MH, Jia Y, Jun H, Lee JH, Hwang KY, Choi DW, Um SJ, Lee BY, You SG, Lee SJ (2012). Taurine is a liver X receptor-α ligand and activates transcription of key genes in the reverse cholesterol transport without inducing hepatic lipogenesis. Mol Nutr & Food Res.

[24] Cooper AD, Chen J, Botelho-Yetkinler MJ, Cao Y, Taniguchi T, Levy-Wilson B (1997). Characterization of hepatic-specific regulatory elements in the promoter region of the human cholesterol 7alpha-hydroxylase gene. J Biol Chem.

[25] Inoue Y, Yu AM, Yim SH, Ma X, Krausz KW, Inoue J, Xiang CC, Brownstein MJ, Eggertsen G, Bjorkhem I, Gonzalez FJ (2006). Regulation of bile acid biosynthesis by hepatocyte nuclear factor 4α. J Lipid Res.

[26] Abrahamsson A, Gustafsson U, Ellis E, Nilsson LM, Sahlin S, Bjorkhem I, Einarsson C (2005). Feedback regulation of bile acid synthesis in human liver: importance of HNF-4alpha for regulation of CYP7A1. Biochem Biophys Res Commun.

[27] Gupta S, Stravitz RT, Dent P, Hylemon PB (2001). Down-regulation of cholesterol 7alpha-hydroxylase (CYP7A1) gene expression by bile acids in primary rat hepatocytes is mediated by the c-Jun N-terminal kinase pathway. J Biol Chem.

[28] De Fabiani E, Mitro N, Anzulovich AC, Pinelli A, Galli G, Crestani M (2001). The negative effects of bile acids and tumor necrosis factor-alpha on the transcription of cholesterol 7alpha-hydroxylase gene (CYP7A1) converge to hepatic nuclear factor-4: a novel mechanism of feedback regulation of bile acid synthesis mediated by nuclear receptors. J Biol Chem.

[29] Das J, Ghosh J, Manna P, Sil PC (2010). Protective role of taurine against arsenic-induced mitochondria-dependent hepatic apoptosis via the inhibition of PKCδ-JNK pathway. PLoS One.

